# Cranial nerve involvement among COVID-19 survivors

**DOI:** 10.3389/fneur.2023.1182543

**Published:** 2023-08-04

**Authors:** Mohammad Eghbal Heidari, Pershang Nazemi, Elham Feizabad, Farzaneh Beiranvand, Mahdieh Afzali

**Affiliations:** ^1^Student‘s Scientific Research Center, Tehran University of Medical Sciences, Tehran, Iran; ^2^Yas Hospital Complex, Tehran University of Medical Sciences, Tehran, Iran; ^3^Community Medicine Specialist, Department of Obstetrics and Gynecology, Yas Hospital, Tehran University of Medical Sciences, Tehran, Iran; ^4^School of Nursing and Midwifery, Tehran University of Medical Sciences, Tehran, Iran; ^5^Department of Neurology, School of Medicine, Yas Hospital, Tehran University of Medical Sciences, Tehran, Iran

**Keywords:** COVID-19, cranial nerve, survived, neurology, infection epidemiology

## Abstract

**Introduction:**

COVID-19 was first reported in November 2019 in China and rapidly spread across the globe. COVID-19 causes neurologic symptoms and complications, which may persist even after recovery in patients. The objective of this research was to determine the involvement of cranial nerves in COVID-19 survivors.

**Method:**

This was a retrospective study. The study was conducted between March and July of 2022. The analysis included 98 patients with a certain positive polymerase chain reaction. SPSS software version 19 was utilized for data analysis.

**Results:**

The average age of the participants was 40.47 years (8.81). The olfactory nerve was found to be the most frequently involved cranial nerve (36.7%). Over 20% of participants had a taste disorder. The findings from the regression analysis indicated that lung involvement and age have a direct and significant relationship with cranial nerve involvement and can serve as its predictors (*p* = 0.001).

**Conclusion:**

It seems that cranial nerve involvement was sustained in COVID-19 patients who survived. In addition, elderly patients and patients with severe illnesses were more likely to show cranial symptoms. It is necessary to monitor COVID-19 survivors for neurological symptoms.

## Introduction

COVID-19 was first reported in November 2019 in Wuhan Province, China. It rapidly spread to other countries, causing the COVID-19 pandemic (1). In Iran, the detection of cases began in March 2020. Coronaviruses are a large family of viruses that has been linked to Middle East respiratory syndrome (MERS-CoV) and severe acute respiratory syndrome (SARS-CoV) (2, 3). The symptoms of COVID-19 vary depending on the underlying disease, age, and immune system condition. According to previous research, the majority of symptomatic and severe cases of COVID-19 occurred in individuals with underlying diseases, such as diabetes, hypertension, and cardiac disease, as well as in the elderly (4, 5).

COVID-19 is characterized by fever, fatigue, cough, myalgia, and headache (6). In some cases, neurological and unusual symptoms have been observed (7–9). Coordination deficit, cognitive impairment, paresis, abnormal reflex status, sensory abnormalities, general muscle weakness and pain, hyposmia, and headache were identified as major clinical neurological characteristics of hospitalized COVID-19 patients by Ermis's study (10). In addition, Nazari's meta-analysis demonstrated that the central nervous system is involved, with headache, vertigo, and impaired consciousness being the most common CNS symptoms of COVID-19 (11).

Important studies on the prevalence of cranial nerve involvement and peripheral nervous system symptoms were found. Some COVID-19 patients have reported olfactory, gustatory, and gastrointestinal issues (12, 13). Several studies have reported peripheral nerve involvement in patients with COVID-19 (14–16). Doblan reported cranial nerve involvement in COVID-19 patients in a study (17). The most frequently involved cranial nerves, according to a study by Finsterer et al., are cranial nerves VII, VI, and III, which manifest as hypogeusia/ageusia, facial palsy, or ophthalmoparesis (16). In another study, patients with COVID-19 exhibited peripheral nervous system (PNS) manifestations including nerve pain and skeletal muscle injury, Guillain–Barré syndrome, cranial polyneuritis, neuromuscular junction disorders, neuro-ophthalmological disorders, neurosensory hearing loss, and dysautonomia (14). Guerrero reported that Guillain–Barré syndrome, facial palsy (Bell syndrome), Miller–Fisher syndrome, polyneuritis cranialis, and other cranial nerve disorders are frequently observed in patients with COVID-19 (15). Evidence shows that many symptoms of the disease may persist after survival.

The majority of previous studies have evaluated neurological symptoms and complications in COVID-19 patients. During the course of the disease, COVID-19 patients exhibit a variety of symptoms, but many of these symptoms disappear after the patient recovers. Nonetheless, evidence suggests that some neurological symptoms may persist in patients and must be evaluated. On the other hand, there are very few studies evaluating neurological symptoms after recovery in COVID-19 patients, and there is a need to investigate peripheral nervous system symptoms. In light of the importance of identifying neurological symptoms in COVID-19 survivors and the paucity of research in this area, we sought to investigate cranial nerve involvement in COVID-19 survivors at Yas Hospital in Tehran.

## Method

This study was a retrospective investigation conducted from March to July 2022. After receiving approval from the Tehran University of Medical Sciences Ethics Committee (IR.TUMS.NI.REC.1400.067), 98 patients with a positive polymerase chain were studied. The inclusion criteria for COVID-19 included a certain PCR. Patients with a history of stroke before and after COVID-19 and patients with a history of mental illness before and after COVID-19 were the exclusion criteria.

The age, sex, height, and weight of the patients, as well as their referral complaints, examination findings, blood and radiological data, and comorbidity, were all measured. Patients' treatment procedures were also documented.

Respecting the confidentiality of the information and ethical principles, the researcher extracted the patients' contact information in each case of COVID-19, contacted the patients, and inquired about the cranial nerves. The questions were specific and related to temporary or permanent cranial nerve disorders caused by COVID-19 infection (Appendix). The incidence of symptoms was reported 2 months after discharge.

## Data analysis

SPSS software version 22 was used to analyze the data. The Kolmogorov–Smirnov test was used to examine the variables' normality. For categorical variables (Frequency), number values were reported. The mean and standard deviation were reported for continuous variables. A *t*-test was utilized to examine the mean difference between groups. In the classification data analysis, Pearson's chi-square and the Fisher test were employed. After adjusting the data for age and sex, a logistic regression analysis was performed to determine the most accurate predictor of cranial nerve involvement. A *p*-value of < 0.05 was considered statistically significant.

## Results

The average age and body mass index (BMI) of the survivors were 40.47 (8.81) and 26.47 (3.20). Participants' mean hospitalization rate was 1.54 (0.50). Of the 98 participants in the study, 54 were female. The incidence of cranial nerve involvement is shown in Table 1. Hypertension was the most prevalent underlying disease among the participants (11.2%). There were 30 patients hospitalized in the intensive care unit, and 28 of those patients were receiving mechanical ventilation; 34 individuals had a history of lung involvement, and 29 individuals received corticosteroids. The olfactory nerve was found to be the most frequently involved cranial nerve (36.7%); 8.2% of participants reported hoarseness, while 11.2% reported blurred vision; 11.2% of individuals reported problems with ptosis, and one individual had diplopia; and 8.2% had difficulty swallowing. More than 20% of the participants had gustatory dysfunction. Tinnitus affected 7.1% of the participants. Facial nerve involvement was not reported among the participants. In addition, none of the participants had abnormal shoulder or tongue movements and chewing problems.

**Table 1:** Frequency distribution of cranial nerve involvement.

**Cranial nerve**	***N***	**%**
Olfactorius	36	36.7
Opticus	11	11.2
Oculomotorius	11	11.2
Trochlearis	1	1
Trigeminus	10	10.2
Abducens	1	1
Facialis	-	-
Vestibulocochlearis	20	20.4
Glossopharyngeus	7	7.1
Vagus	8	8.2
Accessories	8	8.2
Hypoglossus	-	-

The characteristics of cases with and without cranial nerve involvement are presented in Table 2. The age, sex distribution, body mass index, co-morbidities, average length of stay in the hospital, hospitalization in the intensive care unit, COVID-19 diagnosis, pulmonary involvement, and average length of hospitalization were compared between groups with and without cranial nerve involvement. The independent *t*-test shows that the cranial nerves involved vary between age groups (*p* = 0.003).

**Table 2:** Demographic and clinical characteristics of participants according to the groups with and without cranial nerve involvement.

**Variables**	**No cranial nerve involvement, mean ±SD, *N* (%)**	**Cranial nerve involvement, mean ±SD, *N* (%)**	***P*-value**
Age	43.24 ± 10.47	39.31 ± 7.80	0.044
BMI	26.14 ± 2.94	26.61 ± 3.31	0.504
Hospitalization	1.62 ± 0.49	1.50 ± 0.50	0.309
**Sex**			
Male	13 (29.54)	31 (70.46)	0.993
Female	16 (28.60)	38 (70.4)	
Underlying disease	6 (22)	22 (78)	0.263
Without underlying disease	23 (32.8)	47 (67.2)	
Diabetes	2 (22)	7 (88)	0.765
Hypertension	3 (28)	8 (72)	
Lung disease	0	3 (100)	
Renal disease	0	2 (100)	
Asthma	2 (50)	2 (50)	
Lung involvement	16 (47)	18 (53)	0.443
Without lung involvement	22 (32)	47 (68)	
Corton	7 (24)	22 (76)	0.519
Without the use of Corton	43 (33.9)	84 (66.1)	
ICU admission	8 (27)	22 (73)	0.673
Without ICU admission	21 (30)	47 (70)	
Mechanical ventilation	6 (22)	22 (78)	0.263
Without mechanical ventilation	23 (32.8)	47 (67.2)	

The results of the regression analysis indicated that lung involvement and age have a direct and significant relationship with cranial nerve involvement and can serve as its predictors (*p* = 0.001). There was no significant correlation between other variables and cranial nerves (Table 3).

**Table 3:** Results of logistic regression analysis.

**Variables**	**B**	**SE**	**OR**	***P*-value**
Age	−0.51	0.026	0.761	0.049
Male	0.004	0.445	1.103	0.993
BMI	0.047	0.070	0.890	0.500
Underlying disease	0.065	0.372	1.067	0.862
Lung involvement	−1.249	0.463	0.456	0.007
Corton	0.175	0.529	1.210	0.742
ICU	−21.971	28.42	0.213	0.991
Hospitalization	−0.463	0.452	0.321	0.305
Mechanical ventilation	22.417	21.32	0.541	0.991

SE, standard error; OR, odds ratio.

## Discussion

This study revealed that the olfactory nerve was the most defective cranial nerve among COVID-19 survivors (36.7%). The results of the regression analysis indicated that lung involvement and age are predictors of cranial nerve involvement.

Important studies have shown that COVID-19 is a contagious disease with symptoms and complications in the nervous system. COVID-19 has neurological manifestations and central nervous system involvement (8, 9). Nazari's study revealed that headache, dizziness, and impaired consciousness were the most prevalent CNS symptoms of COVID-19 (11). Hyposmia was the most prevalent peripheral manifestation. Regarding the pathophysiology of COVID-19 disease and its relationship with nerves, no specific information is available. SARS-CoV-2 pathophysiology and that of other human coronaviruses shed light on potential neural involvement mechanisms. Similar to other human coronaviruses, SARS-CoV-2 is capable of invading the central nervous system, according to studies (HCoV). Invasion by SARS-CoV-2 is believed to require a cell surface receptor for the binding of the viral spike (S) protein and priming of the S protein by cellular proteases (12, 13).

In this study, the olfactory nerve was involved more than any other cranial nerve. Other manifestations of cranial involvement include hoarseness, hazy vision, ptosis, gustatory dysfunction, and tinnitus. The results are consistent with other research studies (14). According to Finsterer's study, the most frequently involved cranial nerves are cranial nerves VII, VI, and III, which manifest as hypogeusia or azosia, facial paralysis, or ophthalmoparesis. In addition, Andalib's study revealed that cranial polyneuritis, neuromuscular junction disorders, neuro-ocular disorders, sensorineural hearing loss, and dysautonomia were reported as peripheral nervous system (PNS) manifestations in COVID-19 patients (15).

Cranial nerve involvement has also been highlighted in other infectious diseases. In the study by Tsau et al., it was found that among the cranial nerves, the trigeminal nerve, facial nerve, and vestibular nerve were involved in Herpes Zoster infection. Other cranial nerves involved include the glossopharyngeal nerve, vagus nerve, optic nerve, trochlear nerve, and abducens nerve (16). In addition, 38% of patients with tuberculous meningitis demonstrated cranial neuropathy (17). The findings of the articles indicated that cranial nerve involvement is limited not only to COVID-19 individuals but also to people with other infectious disorders. Yamana found that cranial nerve problems, sensory abnormalities, and ataxia were more frequent in the cases after influenza infection than in those after *C. jejuni* infection (46% vs. 15%, 75% vs. 46%, and 29% vs. 4%, respectively) (18). The study by Sharma showed that the abducens nerve was the most frequently involved (32.3%) cranial nerve among affected patients with tuberculous meningitis (17), while the olfactory nerve was the most frequently involved cranial nerve (36.7%) among COVID-19 patients. In other infectious diseases, such as HIV, Bell's palsy was reported in 29% of subjects (19). Our study highlighted that the olfactory nerve was found to be the most frequently involved cranial nerve (36.7%) and more than 20% of the participants had gustatory dysfunction.

Important evidence of cranial involvement has been found in COVID-19 patients. In total, 135 patients were found to have cranial nerve involvement in Doblan's study conducted in Turkey. The olfactory nerve was the nerve that was most affected (20). In addition, this study also revealed that the olfactory nerve was the nerve most commonly affected. Even though this study was conducted on COVID-19 survivors, it yielded comparable results to Doblan's study. The similarity in demographic variables, such as age and sex, as well as underlying diseases and patient diagnoses, is one reason for the similarity between the results of this study and Doblan's study. In a second study conducted in China by Mao, it was determined that 19 patients had peripheral nerve disorders. These conditions affected the taste, vision, and olfactory nerves. Patients with severe disease had a higher incidence of cranial nerve involvement (21). Mao reported that the severity of the disease can result in serious complications in COVID-19 patients (22). In this study, a significant correlation was found between the severity of the disease and cranial nerve involvement. However, additional research is required.

In otolaryngology, the loss of olfactory function due to viral infections is well-known. Viruses, such as rhinovirus, parainfluenza, Epstein–Barr virus, and some coronaviruses, can cause olfactory dysfunction via an inflammatory response in the nasal mucosa and rhinorrhea (23, 24).

Various studies have reported on the prevalence of olfactory disorders in COVID-19 patients. In Yan's study, 59% of patients had olfactory nerve involvement (25). In another study conducted in Spain, the rate of involvement of the olfactory nerve was 32% (26). In Lechien's European study, 85% of patients had a diminished sense of smell (27). There are also numerous accounts of taste disorders. In the French study conducted by Benezit, the prevalence of taste disorders was found to be 93% (28), and 72% of COVID-19 patients in Yan's study in the United States experienced taste disturbances. It appears that taste disorders are more prevalent than smell disorders (29).

The mechanisms involved in olfactory disorders in patients with COVID-19 are still unclear. One such hypothesis is based on the ability of the SARS-CoV-2 virus to cross the blood–brain barrier and enter the brain or that the virus possibly reaches the brain via a hematogenous route. The coronavirus, on the other hand, can enter the brain via the following routes: 1- olfactory nerves; 2- cribriform plaque; or 3- peripheral trigeminal. The second phenomenon can endanger the trigeminal and olfactory nerves and lead to dysosmia and dysgeusia. There may also be other ways that COVID-19 causes anosmia (30–32).

In addition, studies have revealed information regarding the coexistence of taste and smell disorders. Cazzola et al. found that anosmia and taste disturbances are associated with elevated levels of interleukin-6 (IL-6), a key pro-inflammatory cytokine (33). Tham et al. bolster this supposition (34). He hypothesized that the inflammatory cytokine milieu in the nasal cavity, such as in chronic rhinosinusitis, could potentially affect olfactory neuron function. Regarding the causes of taste disorders, numerous mechanisms exist. Due to the intimate relationship between these two chemical sensory systems, the concurrent presence of olfactory disorders may contribute to taste disorders in patients with COVID-19 (30).

The fact that SARS-CoV-2 binds to ACE2 receptors on taste buds at the same time as ACE1 receptors may make taste problems poor. Indeed, Xu and his colleagues investigated the expression of ACE2 in the oral cavity and discovered that ACE2 receptors were sparsely expressed at various sites in the oral cavity, particularly in the tongue more than in oral and gingival tissues. These findings suggest that the oral mucosa may represent a potential high-risk route for COVID-19 transmission (35).

In this study, additional cranial nerves were affected. Vision and chewing disturbances were noted in the findings. In this regard, no specific mechanism has been proposed, and research in this area is limited. However, corticosteroids, immunosuppressants, anticoagulants, cerebral artery thrombosis, and cranial nerve inflammation can all cause cranial disorders (36).

The pathophysiology of cranial nerve involvement is still unknown, but it can be assumed that cranial nerve involvement is caused by virus entry into the intracellular space of neurons at a distal site and retrograde transmission of virus particles to the brain (30). In a study of 43 people who died of COVID-19, SARS-CoV-2 viral proteins were found in nerves coming from the lower brain stem and in brain stem cells, which supports this hypothesis (37). Furthermore, virus particles have been found in neurons and axons of cranial nerves in multiple autopsy studies (38).

Cranial involvement in COVID-19 patients is not uncommon, and significant research in this area has been published. Approximately 36.4% of hospitalized patients had signs of nervous system involvement, such as dizziness, headache, taste disorder, hypoxemia, hemorrhagic and ischemic muscle damage, and brain damage, according to the study by Mao et al. (21). In total, 135 patients exhibited symptoms of cranial nerve involvement, according to a separate study (20). Important case reports have also demonstrated the involvement of the third to ninth cranial nerves in COVID-19 patients (39–41).

This study has both strengths and weaknesses. The evaluation of cranial nerve involvement in COVID-19 survivors is one of the study's strengths and positive aspects. However, this study had some limitations. Examining the cranial nerves through a physical examination by specialists would be superior and more accurate. Examinations by otolaryngologists, neurologists, and ophthalmologists can be useful in the accurate diagnosis of cranial nerve involvement. Another limitation of the study was that the timing of cranial nerve involvement was unknown. In addition, cranial nerve examinations of patients after discharge would have provided more precise results regarding cranial nerve involvement, which was not possible in this study.

## Conclusion

COVID-19 disease typically causes symptoms of cranial nerve involvement. However, it appears that these symptoms may persist after the disease has resolved. Additionally, patients with severe illnesses and elderly people are more likely to exhibit these symptoms. After recovery, patients with severe diseases must be examined and evaluated.

## Data availability statement

The original contributions presented in the study are included in the article/Supplementary material, further inquiries can be directed to the corresponding author.

## Ethics statement

The studies involving human participants were reviewed and approved by research Ethics Committee of Neurological Institute, Tehran University of Medical Sciences. The patients/participants provided their written informed consent to participate in this study.

## Author contributions

Study design: MA, MH, and PN. Data collection: PN, MA, and EF. Data analysis: MA and EF. Study supervision: MA. Manuscript writing: MH, FB, and MA. Critical revisions for important intellectual content: MA and MH. All authors contributed to the article and approved the submitted version.
