# Supplementary material for: Cranial nerve involvement among COVID-19 survivors
Source: Front Neurol. 2023 Aug 4;14:1182543. doi: 10.3389/fneur.2023.1182543 (PMC10436332; doi:10.3389/fneur.2023.1182543)
Supplement: Supplementary file 1 [file Data_Sheet_1.docx]

**Cranial nerve questionnaire**

| 1. Have you lost your sense of smell temporarily or permanently? yes No |
| --- |
| 1. 2- Have you temporarily or permanently lost your vision or do you experience blurred vision? yes No |
| 1. 3-Do your eyelids droop temporarily or permanently? yes No |
| 1. Are your eye movements limited temporarily or permanently, or do you suffer from double vision? yes No |
| 1. Is your face numbed temporarily or permanently? yes No |
| 1. Do you experience difficulty chewing? yes No |
| 1. Are your facial movements limited or reduced temporarily or permanently (such as facial stiffness, eye closure disorder, facial muscle weakness, etc )؟ yes No |
| 1. Have you had permanent or temporary difficulty tasting food? yes No |
| 1. Have you experienced ringing in the ears, the sensation of objects rotating around you, and temporary or permanent hearing loss? yes No |
| 1. - Is your voice affected by violence temporarily or permanently? yes No |
| 1. - Have you ever had difficulty temporarily or permanently swallowing food? yes No |
| 1. Do you experience temporary or permanent difficulty turning your head or lifting your shoulders? yes No |
| 1. Do you have temporary or permanent difficulty moving your tongue? yes No |
